# Genome-Wide Analysis and Expression Profiling of the Phospholipase C Gene Family in Soybean (*Glycine max*)

**DOI:** 10.1371/journal.pone.0138467

**Published:** 2015-09-30

**Authors:** Fawei Wang, Yu Deng, Yonggang Zhou, Jinye Dong, Huan Chen, Yuanyuan Dong, Nan Wang, Xiaowei Li, Haiyan Li

**Affiliations:** College of Life Sciences, Engineering Research Center of the Chinese Ministry of Education for Bioreactor and Pharmaceutical Development, Jilin Agricultural University, Changchun, China; The Chinese University of Hong Kong, HONG KONG

## Abstract

Phosphatidylinositol-specific phospholipase C (PI-PLC) hydrolyses phosphatidylinositol-4,5-bisphosphate to produce diacylglycerol and inositol 1,4,5-trisphosphate. It plays an important role in plant development and abiotic stress responses. However, systematic analysis and expression profiling of the phospholipase C (PLC) gene family in soybean have not been reported. In this study, 12 putative PLC genes were identified in the soybean genome. Soybean PLCs were found on chromosomes 2, 11, 14 and 18 and encoded 58.8–70.06 kD proteins. Expression pattern analysis by RT-PCR demonstrated that expression of the *GmPLCs* was induced by PEG, NaCl and saline-alkali treatments in roots and leaves. *GmPLC* transcripts accumulated specifically in roots after ABA treatment. Furthermore, *GmPLC* transcripts were analyzed in various tissues. The results showed that *GmPLC7* was highly expressed in most tissues, whereas *GmPLC12* was expressed in early pods specifically. In addition, subcellular localization analysis was carried out and confirmed that *GmPLC10* was localized in the plasma membrane in *Nicotiana benthamiana*. Our genomic analysis of the soybean PLC family provides an insight into the regulation of abiotic stress responses and development. It also provides a solid foundation for the functional characterization of the soybean PLC gene family.

## Introduction

The PLC (phospholipase C) gene family is a subfamily of the phospholipase superfamily, which includes phospholipase D, phospholipase C, phospholipase A_1_ and phospholipase A_2_. PLCs are divided into phosphatidylinositol-specific phospholipase C (PI-PLC) and non-specific phospholipase (NPC) by different hydrolysis sites [1]. NPC hydrolyzes the membrane lipids phosphatidylcholine and phosphatidylethanolamine in a calcium-independent manner [1]. It plays an important role in the supply of inorganic phosphate in the root plasma membrane during phosphate deprivation [2]. PI-PLC, also named PLC, exists in both animals and plants. PLC-catalyzed production of the second messengers inositol 1,4,5-trisphosphate (IP_3_) and diacylglycerol (DAG) is a key event in the transduction of agonist-dependent signals over the plasma membrane [3, 4]. IP_3_ was shown to release Ca^2+^ from internal stores to the cytoplasm in animals. However, an IP_3_ receptor has not been found in plants. Furthermore, protein kinase C (PKC), which is the target of DAG in animals, is lacking from plant genomes [5]. In plants, DAGs can be phosphorylated by DAG kinase (DGK) and converted into phosphatidic acids (PAs), which have emerged as a new class of lipid mediators involved in diverse cellular functions in plants [6]. IP_3_ is rapidly converted into IP_6_ and causes the release of Ca^2+^ in plants [7]. The function of PLC is carried out mainly by the PA and IP_6_ pathways in plants.

Two different types of PI-PLC enzymes, soluble and membrane-bound, were identified in the cells of several plants in early studies [8–13]. Only membrane-bound PI-PLCs were characterized to hydrolyze phosphatidylinositol-4,5-bisphosphate (PI(4,5)P_2_) and phosphatidylinositol 4-phosphate (PI4P) [14], although the activities of these PI-PLCs were measured from crude protein extracts. Nonetheless, in vitro PLC activity has been confirmed for recombinant Arabidopsis PLCs. All five AtPLC fusion proteins tested required micromolar levels of Ca^2+^ for PI(4,5)P_2_ hydrolyzing activity with the exception of AtPLC4 [15]. However, our research showed that the activity of OsPLC1 was inhibited under high Ca^2+^ concentrations (more than 1 mM) (data not shown). This should be caused mainly by feedback in the PLC signal pathway. Two messengers produced by PLC are thought to play signaling roles in guard cells [7], pathogen responses, Nod factor signaling and carbon fixation in C4 plants [16–18]. In Arabidopsis, there are nine known PI-PLC sequences in the genome [19]. Pokotylo et al. [20] characterized six NPCs in Arabidopsis and NPC involvement has been identified in the regulation of diverse cellular processes including root development and hormone signaling [21], Al stress signaling [22], abscisic acid (ABA) sensing, and tolerance to hyperosmotic and salt stresses [1, 23]. Both PI-PLCs and NPCs have been found and characterized in the rice and tomato genomes [24, 25].

Soybean PLC was first cloned and characterized in 1995 [26]. Soluble enzyme fractions that hydrolyzed phosphoinositides were separated from soybean sprouts using Matrex green gel column chromatography [27]. Functional analysis of soybean PLC was carried out using the PI-PLC specific inhibitor U-73122 and suggested that PI-PLC may negatively regulate the expression of defense genes [28]. In this study, we identified and characterized 12 PLC genes in the soybean genome. The expression profiles of soybean PLC genes were examined in different tissues and under various abiotic stresses. Moreover, subcellular localization analysis was carried out and showed that GmPLC10 was located in the plasma membrane.

## Materials and Methods

### Genomic search and sequence analysis

We searched for soybean PLC genes in the Phytozome v9.1 database (http://www.phytozome.org/). The key words “phospholipase C” were also searched using the browse tool on the NCBI website (www.ncbi.nlm.nih.gov/). The conserved domains of GmPLC proteins were identified by CDSEARCH/cddv3.13 (http://www.ncbi.nlm.nih.gov/Structure/cdd/wrpsb.cgi). Arabidopsis PLC sequences were obtained from TAIR 10.0 (The Arabidopsis Information Resource). Rice PLC sequences were obtained from RGAP (Rice Genome Annotation Project). The loci of PLCs from Arabidopsis and rice are shown in S1 Table. A phylogenetic tree was constructed by MEGA5.1 with the default parameters [29].

### Plant growth and abiotic stress treatment

Soybean (*Glycine max*, Williams 82) seeds were grown in the natural environment on 5 May of 2014 at Jilin Agricultural University, Changchun, Jilin, China. The soil type is black soil and the row spacing is 30 cm. For expression analysis of *GmPLCs* in various tissues, leaves, roots and stems were harvested 30 d after sowing. Flowers were harvested about 80 d after sowing. Early and late pods were harvested 7 and 30 d after flowering. Immature and mature seeds were collected at 20 and 40 d after flowering. In addition, soybean seeds were grown in hydroponic pots that contained Hoagland’s solution under a 12 h light/12 h dark cycle, a constant temperature of 25°C, and a humidity of 70%. The concentrations of Hoagland’s solution for each element are 210 ppm N, 235 ppm K, 200 ppm Ca, 31 ppm P, 64 ppm S, 48 ppm Mg, 0.5 ppm B, 0.5 ppm Mn, 0.05 ppm Zn, 0.02 ppm Cu, 0.01 ppm Mo and 1 to 5 ppm Fe. The nutrient solution was replaced every 2 d. When the *G*. *max* seedlings were at the four-leaf stage (about 10 d), they were transferred into various stress solutions, salt (110 mM NaCl), alkali (100 mM NaHCO_3_), saline-alkali (70 mM NaCl + 50 mM NaHCO_3_), PEG (8% PEG8000) or ABA (100 μM ABA), for 0, 1, 3, 6, 9 and 12 h. Control plants were grown in Hoagland’s solution. The roots and leaves were collected at about 10 AM and stored at −80°C until further use.

### Extraction of total RNA and cDNA synthesis

Total RNA was isolated from the collected tissue samples separately using Trizol reagent (Invitrogen, Carlsbad, CA, USA) following the manufacturer’s protocols. The quality and quantity of the total RNA were determined using a NanoDrop 2000 (ThermoFisher Scientific, Beijing, China). cDNA was synthesized from 2 μg of total RNA using the PrimeScript RT reagent kit (Takara, Japan).

### Quantitative real-time PCR

Gene-specific primers for the *GmPLCs* and reference genes were designed using Primer Express and are shown in S2 Table [30]. For expression analysis under abiotic stresses, *Actin11* was selected as the reference gene. *EF1A* was chosen for the expression analysis in different tissues. RT-PCR was performed on a Stratagene Mx3000P thermocycler (Agilent) with the following program: 95°C for 15 s, followed by 40 cycles of 95°C for 15s and annealing at 60°C for 30 s. The expression level was calculated using the 2^−ΔΔCt^ method for abiotic stress treatments and the 2^ΔCt^ method for different tissues. Triplicates of each reaction were performed.

### Promoter analysis

The promoters of the *GmPLCs*, which were 1 kb upstream from the translation start site, were mapped in the soybean genome. The *cis*-acting regulatory elements were identified in each promoter. Among these, we chose abiotic stress response and development related elements for expression analysis.

### *Nicotiana benthamiana* transient transformation and subcellular localization

For subcellular localization analysis of soybean PLC, *GmPLC10* was introduced into the binary expression vector pCAMBIA1302. The expression vector was transformed into *Agrobacterium tumefaciens* cells of the EHA105 strain. The transient transformation of *N*. *benthamiana* leaves was carried out following Marion et al. [31], with minor revision. For confocal microscopy, tobacco leaves were imaged using an inverted TCS-SPE spectral confocal laser scanning microscope (Leica, Germany). GFP fluorescence was excited with 488 nm argon laser lines with an emission band of 495–540 nm. Triplicate tobacco infiltrations were performed for the subcellular localization experiment.

## Results and Discussion

### Identification of PLC genes in soybean

The genome of soybean was sequenced recently [32]. We searched for soybean phospholipase C in both the genome and GenBank. Twelve PLCs were identified after removing redundant sequences. We named these 12 PLC genes *GmPLC1* to *GmPLC12* according to their physical locations on chromosomes 1–20 (Table 1). There are four, six and nine PLCs in the rice, tomato and Arabidopsis genomes, respectively [24, 25, 33]. The greater number of PLCs in soybean implied the importance and complexity of GmPLCs. The soybean PLCs were spread over chromosomes 2, 11, 14 and 18 equally (S1 Fig). Interestingly, all three PLC genes in each chromosome were located adjacent to one another, with the exception of *GmPLC9* in chromosome 14. A similar phenomenon was found in Arabidopsis; *AtPLC1*, *AtPLC4* and *AtPLC5* are located in a 12 kb fragment on chromosome 5 [33]. The open reading frames (ORFs) of the soybean PLC genes ranged from 1623 to 1833 bp and encoded proteins of 540 to 610 amino acids. The theoretical isoelectric points of the soybean PLC proteins ranged from 5.57 to 9.11 with molecular masses ranging from 58.80 to 70.06 kD. The average length of PLC proteins is about 600 amino acids in plants. The smallest PLC is OsPLC2 from rice, which is composed of 491 residues [24]. In soybean, GmPLC5 and GmPLC11 were smaller than the others (Table 1). Furthermore, the coding sequences were analyzed and we found that all of the soybean PLC genes contained nine exons, except *GmPLC4* and *GmPLC12*. The number of exons in the soybean PLCs was greater than that in rice and Arabidopsis (S2 Table).

**Table 1:** List of 12 PLC genes identified in soybean.

Name	Gene Identifier	Chr.	Location coordinates (5'-3')	ORF length (bp)	Protein			Exons
					Length (aa.)	pI	Mol. Wt. (kD)	
GmPLC1	Glyma02g42410	2	47442411–47446810	1833	610	6.59	70.06	9
GmPLC2	Glyma02g42420	2	47450458–47455299	1713	570	8.44	65.00	9
GmPLC3	Glyma02g42430	2	47463027–47467886	1803	600	5.70	58.80	9
GmPLC4	Glyma11g35290	11	36978149–36982608	1791	596	9.11	68.56	10
GmPLC5	Glyma11g35300	11	36984672–36990916	1623	540	6.01	61.17	9
GmPLC6	Glyma11g35310	11	36993479–36998177	1779	592	5.57	67.29	9
GmPLC7	Glyma14g06450	14	4706397–4711170	1803	600	5.73	68.70	9
GmPLC8	Glyma14g06460	14	4715979–4720795	1767	588	6.57	66.83	9
GmPLC9	Glyma14g37290	14	46572314–46578539	1731	576	6.42	66.38	9
GmPLC10	Glyma18g03090	18	2033312–2038120	1779	592	5.90	67.56	9
GmPLC11	Glyma18g03100	18	2042085–2047648	1671	556	5.70	63.22	9
GmPLC12	Glyma18g03110	18	2048710–2053428	1815	604	8.83	68.99	10

### *GmPLC* domain analysis and phylogenetic analysis

**Fig 1:**
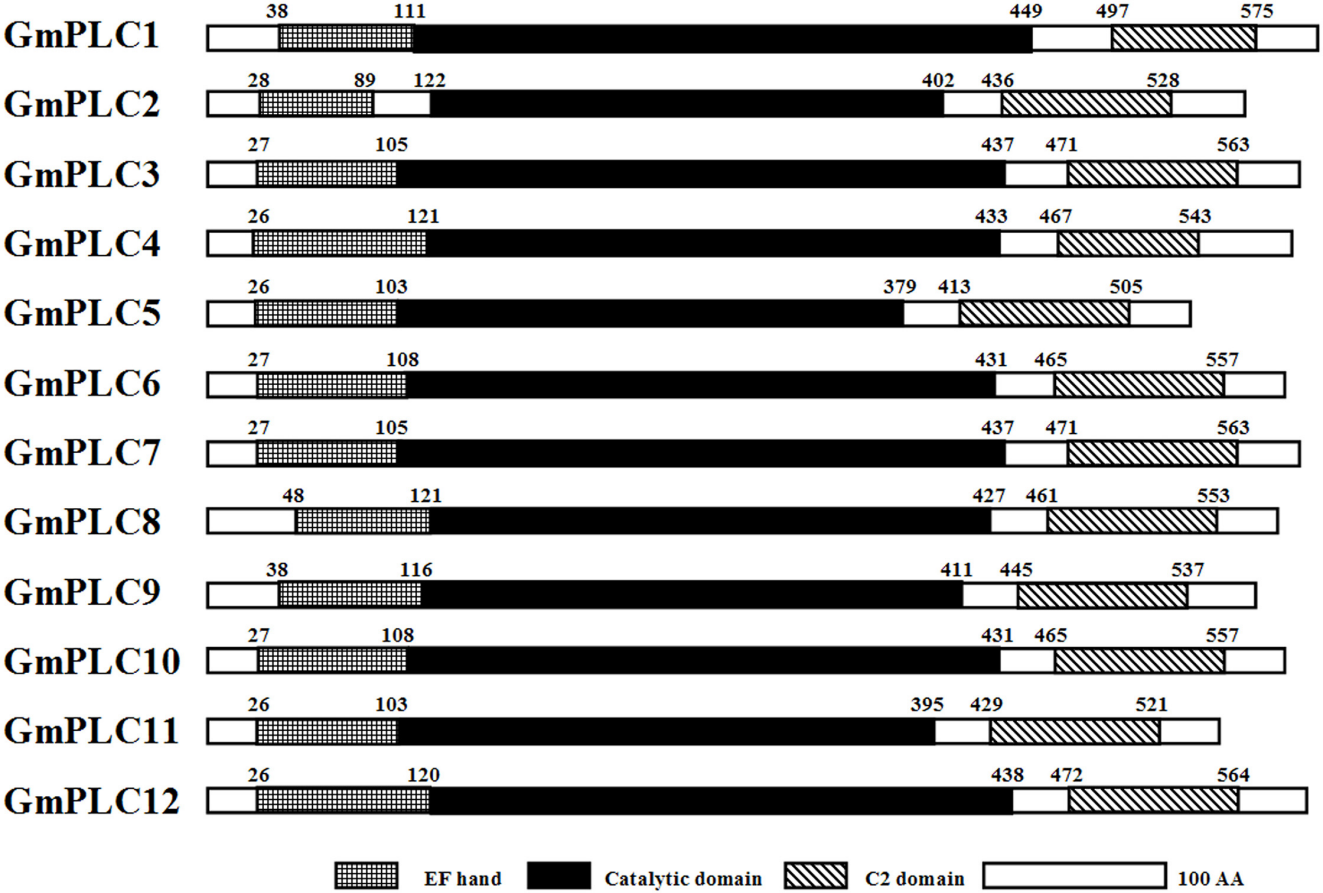
Domain analysis of soybean PLCs.

To characterize the soybean PLC genes, their domain structure was analyzed and a phylogenetic tree was constructed. PLC proteins contain a Pleckstrin Homology (PH) domain, EF hand, catalytic domain and C2 domain in animals. Some also contain a PDZ binding motif, Ras-binding domain, guanine-nucleotide-exchange factor for the Ras domain and Src homology domain [5]. However, in plants, such as soybean, PLCs only contain an EF hand, catalytic domain and C2 domain (Fig 1). The EF hand, which also found in DGK, has a key role in Ca^2+^ binding [34]. The catalytic domain, which contains X and Y domains, is conserved among eukaryotic PLCs [35], especially soybean PLC (S2 Fig). The C2 domain can bind Ca^2+^ and other effectors, including phospholipids, inositol phosphates, and proteins [36–38]. The C2 domain appears at the beginning of phospholipase D [39], but at the end of soybean PLC (Fig 1).

**Fig 2:**
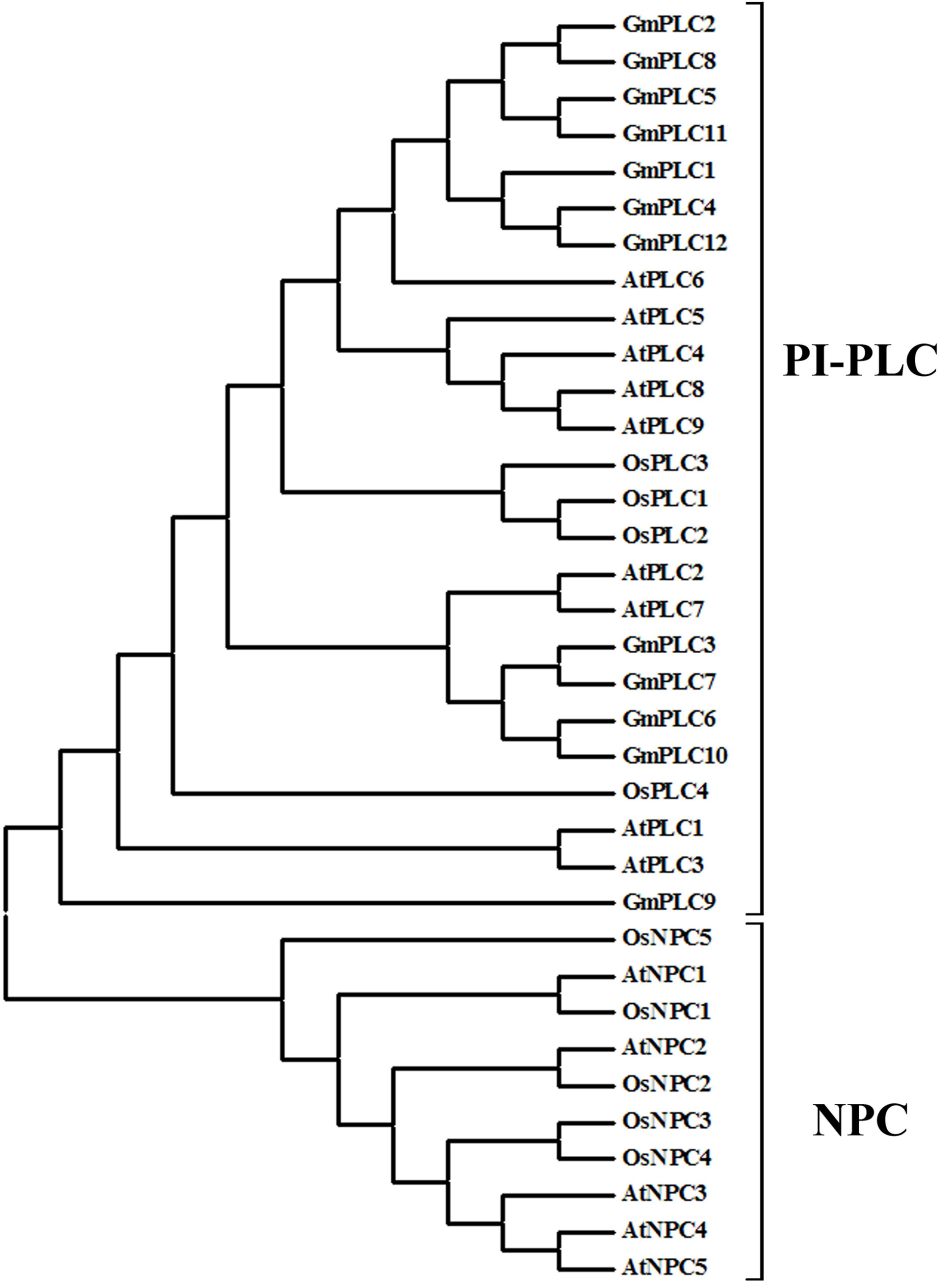
Phylogenetic analysis of the PLC family in soybean, Arabidopsis and rice.

There are two types of phospholipase C (PI-PLC and NPC) in plants. However, no NPCs were found in the soybean genome. Phylogenetic analysis was carried out on the plant PLC and NPC gene families in Arabidopsis, rice and soybean. Soybean PLCs showed high homology to PI-PLCs from Arabidopsis and rice. On the other hand, OsNPCs and AtNPCs shared similarity with each other (Fig 2). The differences between the PLC types derived from diversity in structure and amino acid composition. These results suggest that soybean PLC genes belong to the PLC gene family and should be classified as PI-PLCs.

### Expression analysis of *GmPLCs* under abiotic stresses

**Fig 3:**
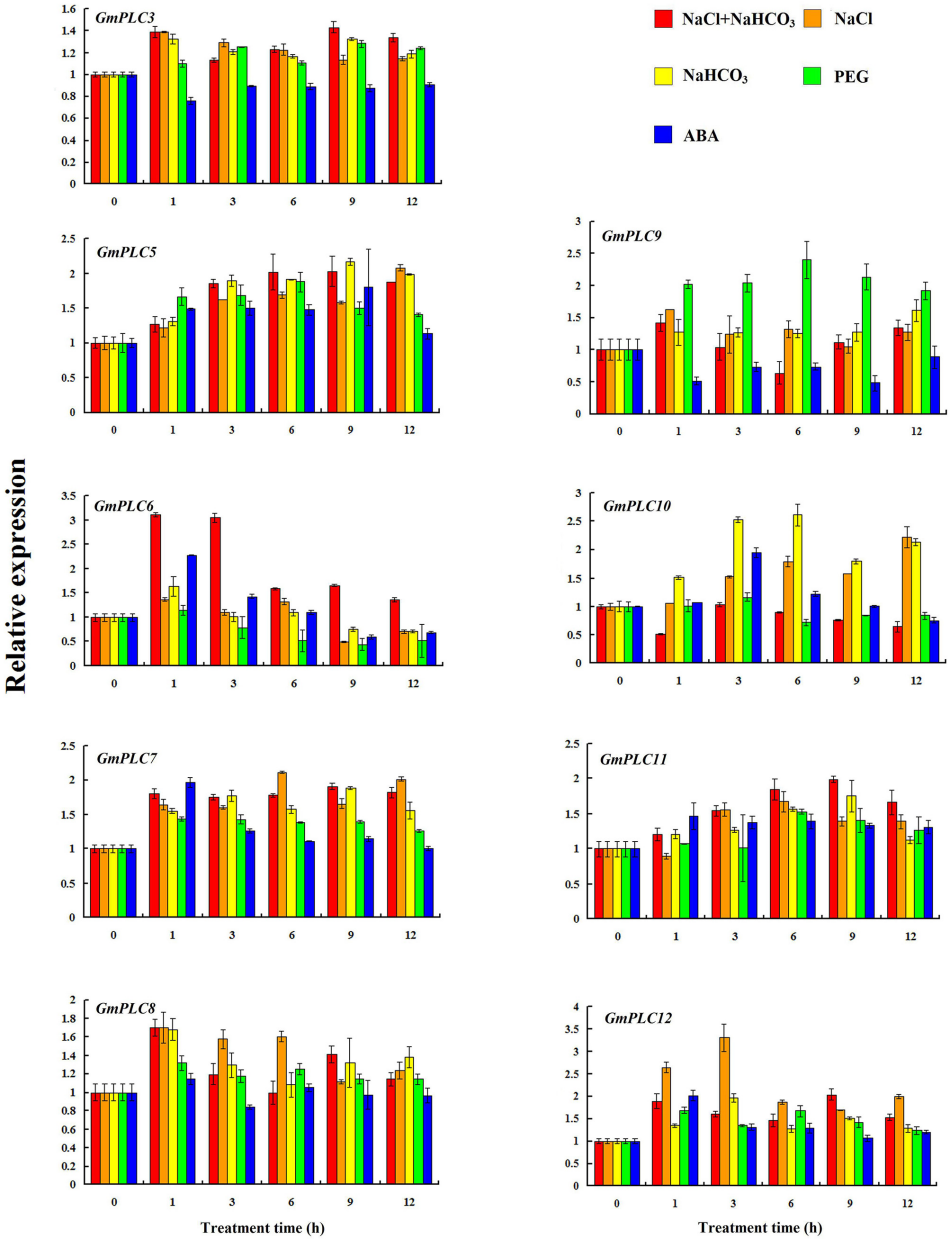
Expression of soybean *PLCs* in leaves under abiotic stress. The expression levels of soybean *PLCs*, which were shown in Y-axis, were compared with the control (0 h). Please note that the expression levels of different PLCs are scaled differently.

To investigate the expression patterns of the soybean PLC genes, qRT-PCR was used to detect their transcript levels in leaves and roots under PEG, ABA, salt, alkali and saline-alkaline treatments. Our result indicated that the expression of *GmPLC1*, *GmPLC2*, *GmPLC4* in leaves and *GmPLC2*, *GmPLC4*, *GmPLC8* in roots cannot be detected. In leaves, *GmPLC6* transcripts accumulated dramatically under saline-alkali and ABA treatments (Fig 3). In pea leaves, *PsPLC* transcription was also stimulated after 30 min induction by ABA [40]. It has been reported that PLC plays a role in the events associated with the inhibition of stomatal opening by ABA [41]. This implies that PLC takes part in the regulation of drought stress in plants. On the other hand, the expression levels of *GmPLC9* were induced by PEG stress, specifically. Transcript levels of *GmPLC10* were increased after alkali treatment. After induction by salt, *GmPLC12* transcript levels were more than 3-fold greater than in the control (Fig 3). Recently, *TaPLC1* was characterized to have biological functions in regulating seedling growth and responses to drought and salinity stress [42]. The expression of *TaPLC1* and *AtPLC5* could be induced by salt and PEG treatments [15, 42]. The overexpression of *AtPLC9* improved thermotolerance in Arabidopsis [43]. These results show that plant PLCs play a positive role in abiotic stress responses. However, the expression levels of *GmPLC3* and *GmPLC9* decreased under ABA treatment, and *GmPLC6* transcripts were decreased under PEG treatment (Fig 3). This phenomenon was also observed in rice, in which *OsPLC4* transcripts decreased during salt, cold and drought stresses [24]. It was also shown that one family member had dominant negative effects on the function of the others in Arabidopsis [44]. It is essential to investigate the mechanism by which PLCs function in the regulation of plant abiotic stress responses.

**Fig 4:**
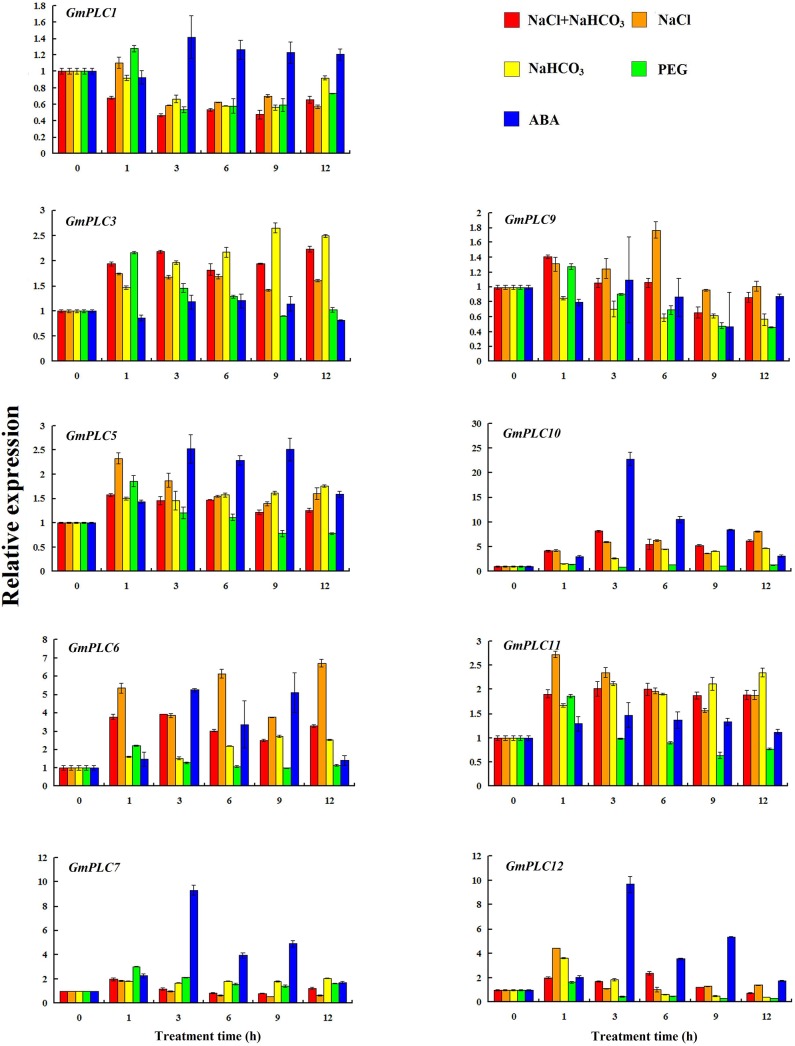
Expression of soybean *PLCs* in roots under abiotic stress. The expression levels of soybean *PLCs*, which were shown in Y-axis, were compared with the control (0 h). Please note that the expression levels of different PLCs are scaled differently.

PA reportedly binds to a number of proteins that play a role during water limiting conditions, such as drought and salinity stress, and has been shown to play an important role in maintaining root system architecture [45]. In addition, DAG, which is a product of PLC, can be subsequently phosphorylated to PA by DGK. Therefore, the expression profiles of the soybean PLC genes were also analyzed in roots. The expression of *GmPLC1* decreased under salt, saline-alkali, alkali and PEG treatments in roots. *GmPLC3*, *GmPLC5*, *GmPLC6*, *GmPLC10*, *GmPLC11* and *GmPLC12* transcripts were induced by salt stress (Fig 4). The expression levels of *GmPLC3*, *GmPLC5*, *GmPLC6*, *GmPLC10*, *GmPLC11* and *GmPLC12* were increased under alkali treatment. *GmPLC3*, *GmPLC5*, *GmPLC6*, *GmPLC10* and *GmPLC11* were expressed under saline-alkaline treatment. ABA treatment stimulated the transcription of *GmPLC5*, *GmPLC6*, *GmPLC7*, *GmPLC10* and *GmPLC12*, especially *GmPLC7*, *GmPLC10* and *GmPLC12* (Fig 4). Although the expression profiles of PLCs in roots have not been analyzed before, PLC signaling has been characterized in the activation of plasmolysis in root cells [46]. Moreover, most of the soybean PLC genes were induced by abiotic stress, especially ABA treatment. This suggests that soybean PLC genes are involved in various stress pathways.

### Expression analysis of *GmPLCs* in various organs

**Fig 5:**
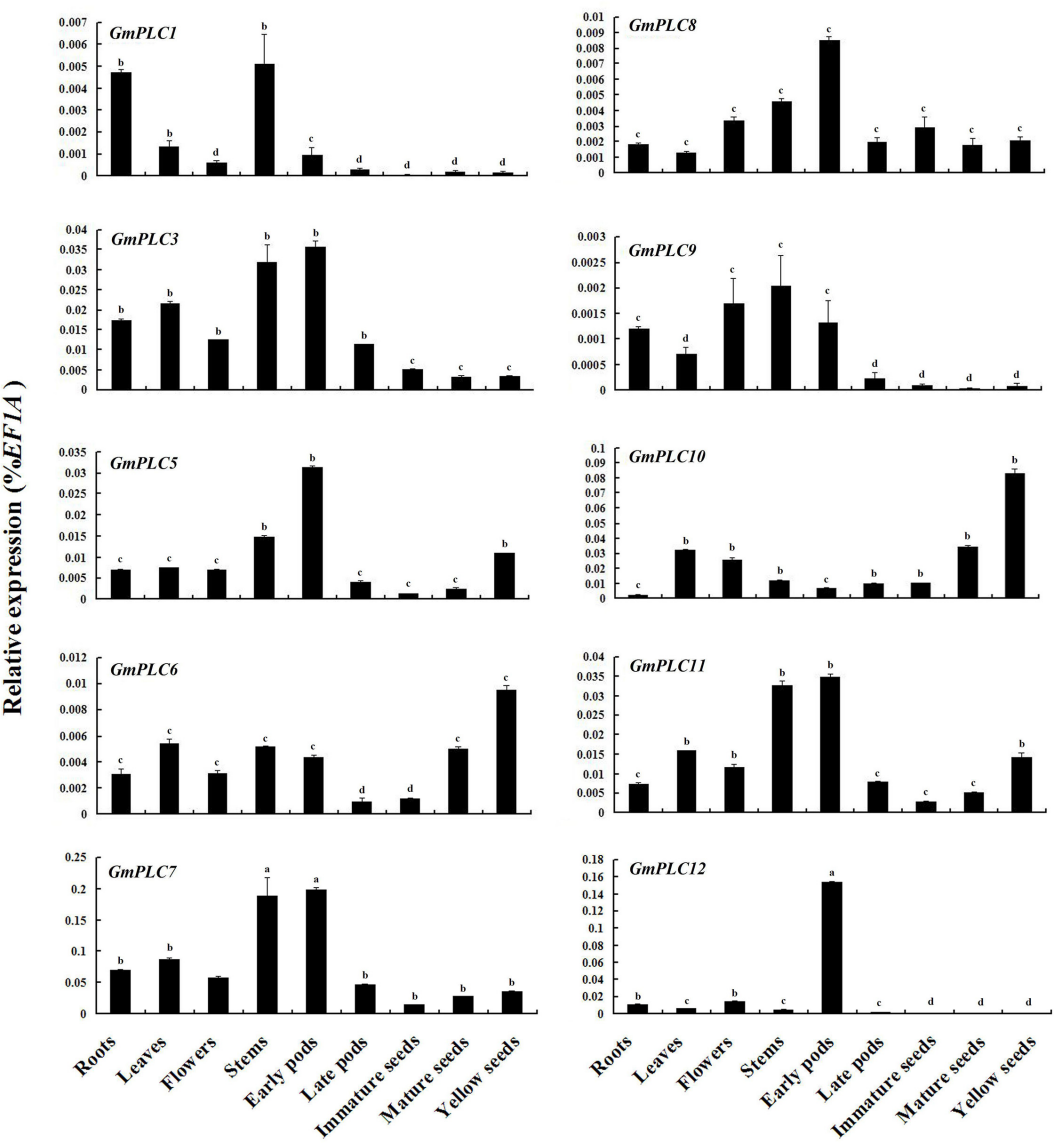
Expression of *GmPLCs* in various organs. The expression levels of soybean *PLCs* was compared with *EF1A* which the expression level of *EF1A* was defined as “1”. a: expression level > 0.1; b: expression level from 0.01 to 0.1; c: expression level from 0.001 to 0.01; d: expression level < 0.001. Please note that the expression levels of different PLCs are scaled differently.

Plant PLC members have been characterized in various cellular processes and signaling networks, and are triggered in response to developmental events in different plant species [47]. To investigate the steady-state expression patterns of the PLC genes in soybean, qRT-PCR was used to detect transcript levels in various organs. High transcript levels of *GmPLC7* were detected in all organs, especially stems and early pods. *GmPLC12* expression was found in early pods specifically, although low transcript levels were found in other organs (Fig 5). In Arabidopsis, *AtPLC2* was highly expressed in leaves, stems, roots and flowers [33]. Recently, microarray analysis of the Arabidopsis PI-PLC gene family was carried out and confirmed that *AtPLC2* transcripts were highly expressed in all organs [48]. *GmPLC3*, *GmPLC5*, *GmPLC10* and *GmPLC11* had medium transcript levels in all organs. The expression levels of *GmPLC1*, *GmPLC6*, *GmPLC8* and *GmPLC9* were lower than the others (Fig 5). In rice, *OsPLC1* and *OsPLC3* were highly expressed in all organs, but lower expression of *OsPLC2* was detected in various organs [24]. The varied expression patterns of the soybean PLC gene family imply that *GmPLCs* are involved in different stages during soybean development.

### *Cis*-regulatory elements in the promoters of soybean PLC genes

**Fig 6:**
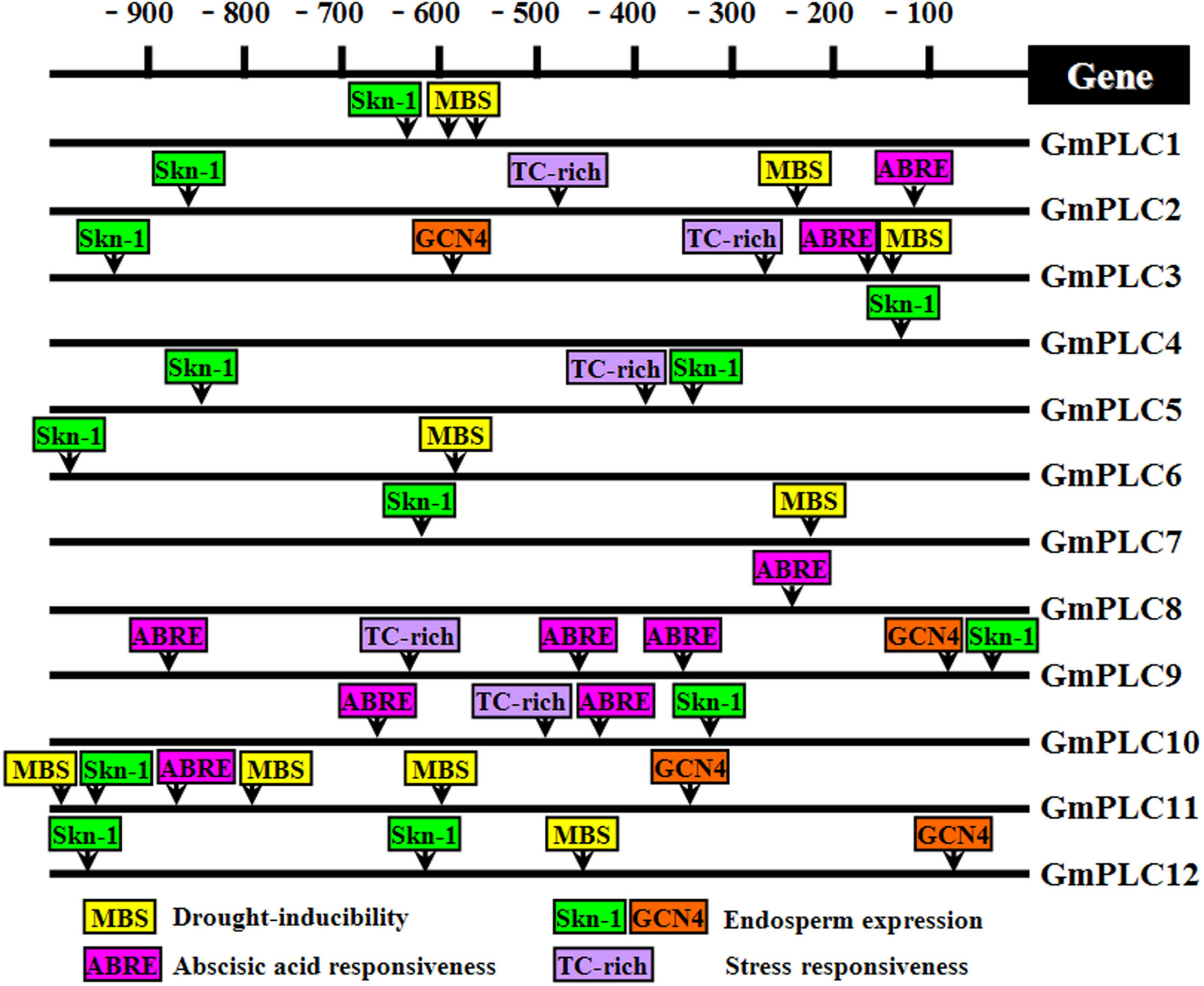
*Cis*-regulatory elements in the promoters of the soybean PLC gene family. The values of -900 to -100 represent the upstream region (from translation start site) of the promoter of all the PLC genes. Various elements such as ABRE, MBS, TC-rich, Skn-1 and GCN4 are present in the promoter.

Gene expression can be regulated at several levels, such as transcription and post-translational modification. The promoter is the region of DNA that initiates the transcription of a particular gene. *Cis*-regulatory elements are functional DNA sequences that precisely control the temporal and spatial expression patterns of the genes expressed in higher plants [49]. In this study, soybean PLC genes were mapped on the genome and their promoter sequences were obtained. The *cis*-regulatory elements, such as MBS (MYB binding site), ABRE (abscisic acid responsive element), TC-rich, Skn-1 and GCN4, were analyzed. These *cis*-regulatory elements are known to regulate various stress responses and plant development [50–53]. Each soybean PLC gene had 1–6 *cis*-regulatory elements in its promoter region (Fig 6). The promoter of *GmPLC10*, which was highly expressed in all organs and induced strongly by abiotic stresses, contained one TC-rich and Skn-1, and two ABRE motifs. *GmPLC3*, *GmPLC11* and *GmPLC12* had more *cis*-regulatory elements, which might account for their responsiveness to abiotic stresses and development (Fig 6, S3 Table). Surprisingly, six *cis*-regulatory elements were found in the promoter of *GmPLC9*, but *GmPLC9* transcripts were low in all organs and insensitive to nearly all abiotic stresses. This is in agreement with previous data on *Glu-A1-1*, *-B1-1* and *-B1-2* [54]. This result implies that other mechanisms modulate the expression of *GmPLC9*.

### Subcellular localization of *GmPLC10*

**Fig 7:**
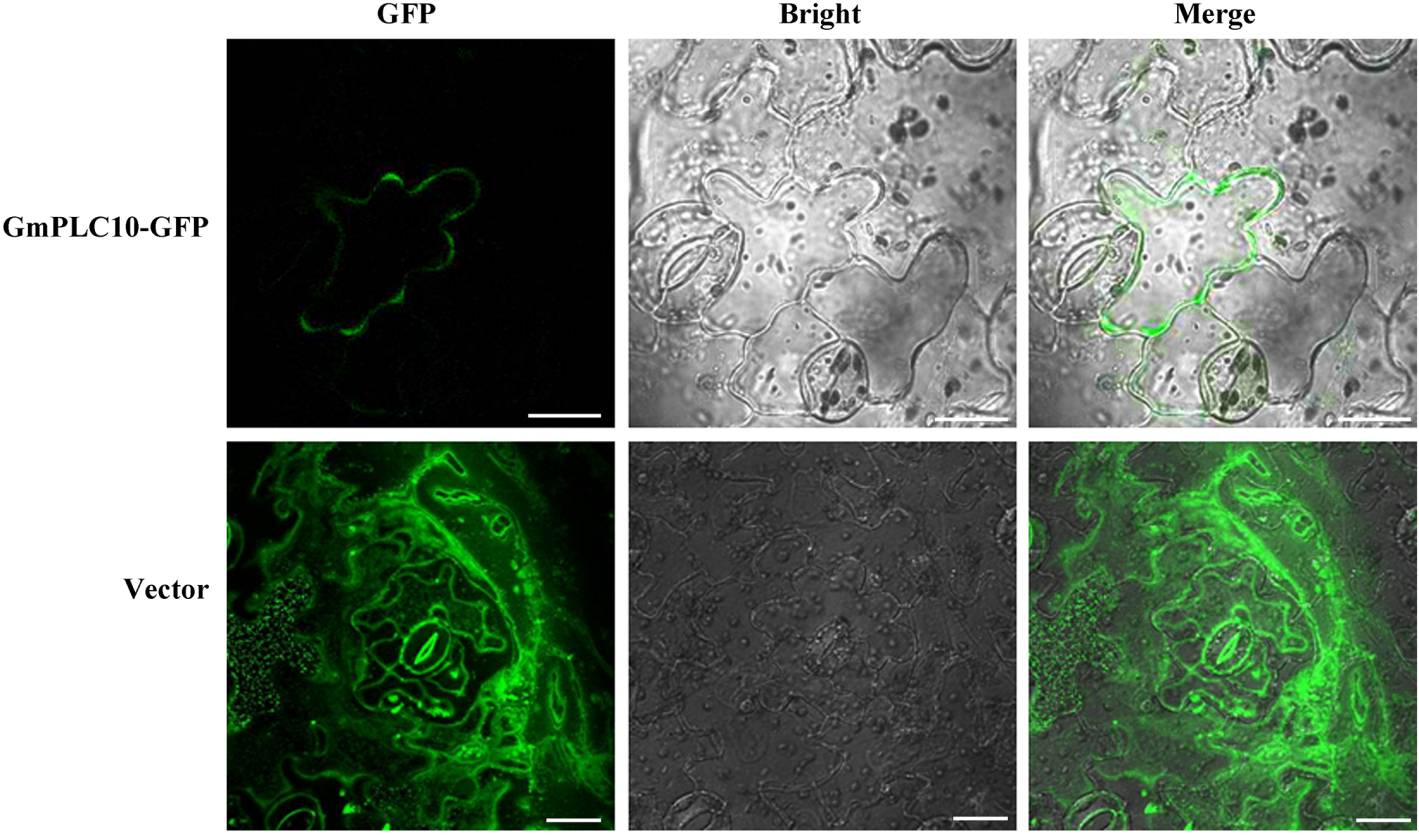
Subcellular localization of *GmPLC10* in *Nicotiana benthamiana* cells. Bar = 20 μm.

Previously, it was reported that the substrates of PLC, PI4P and PI(4,5)P_2_, are located in the plasma membrane [55]. To analyze the location of soybean PLC proteins, GmPLC10 was selected for subcellular localization analysis. *GmPLC10* was fused to the N-terminus of GFP and transformed into *N*. *benthamiana* transiently. In confocal observation, GmPLC10-GFP fluorescence was located in the plasma membrane, whereas fluorescence of the vector control was observed everywhere (Fig 7). This result is consistent with research on AtPLC9 [43]. However, Singh et al. [24] reported that OsPLC1 and OsPLC4 were distributed throughout the cytoplasm and nucleus. The diverse localization of plant PLCs implies that they might take part in different cellular processes during development and abiotic stress.

## Conclusions

In conclusion, we identified 12 GmPLC genes from the soybean genome in this study. Conversed domain and phylogenetic analyses were carried out and verified that all soybean PLC genes belong to the PI-PLC subfamily. The expression profiles of the soybean PLC genes were analyzed during development and various stresses. Together with *cis*-regulatory elements analysis, our results suggest that soybean PLCs are involved in various cellular processes. Moreover, subcellular localization analysis confirmed that GmPLC10 was located in the plasma membrane. Taken together, these results suggest that soybean PLCs is important for plant development and adaptation to environmental stress conditions.

## Supporting Information

S1 Table Loci of PLCs from Arabidopsis and rice. (XLS)

S2 Table Primers used for RT-PCR. (XLS)

S3 Table Numbers of *cis*-regulatory elements in the promoters and expression profiling statistics. (XLS)

S1 Fig Location of PLCs in the soybean chromosomes. (TIF)

S2 Fig Multiple sequence alignment of GmPLCs. (TIF)
