# Supplementary figures and images for: Genome-Wide Analysis and Expression Profiling of the Phospholipase C Gene Family in Soybean (Glycine max)
Source: PLoS One. 2015 Sep 30;10(9):e0138467. doi: 10.1371/journal.pone.0138467 (PMC4589352; doi:10.1371/journal.pone.0138467)

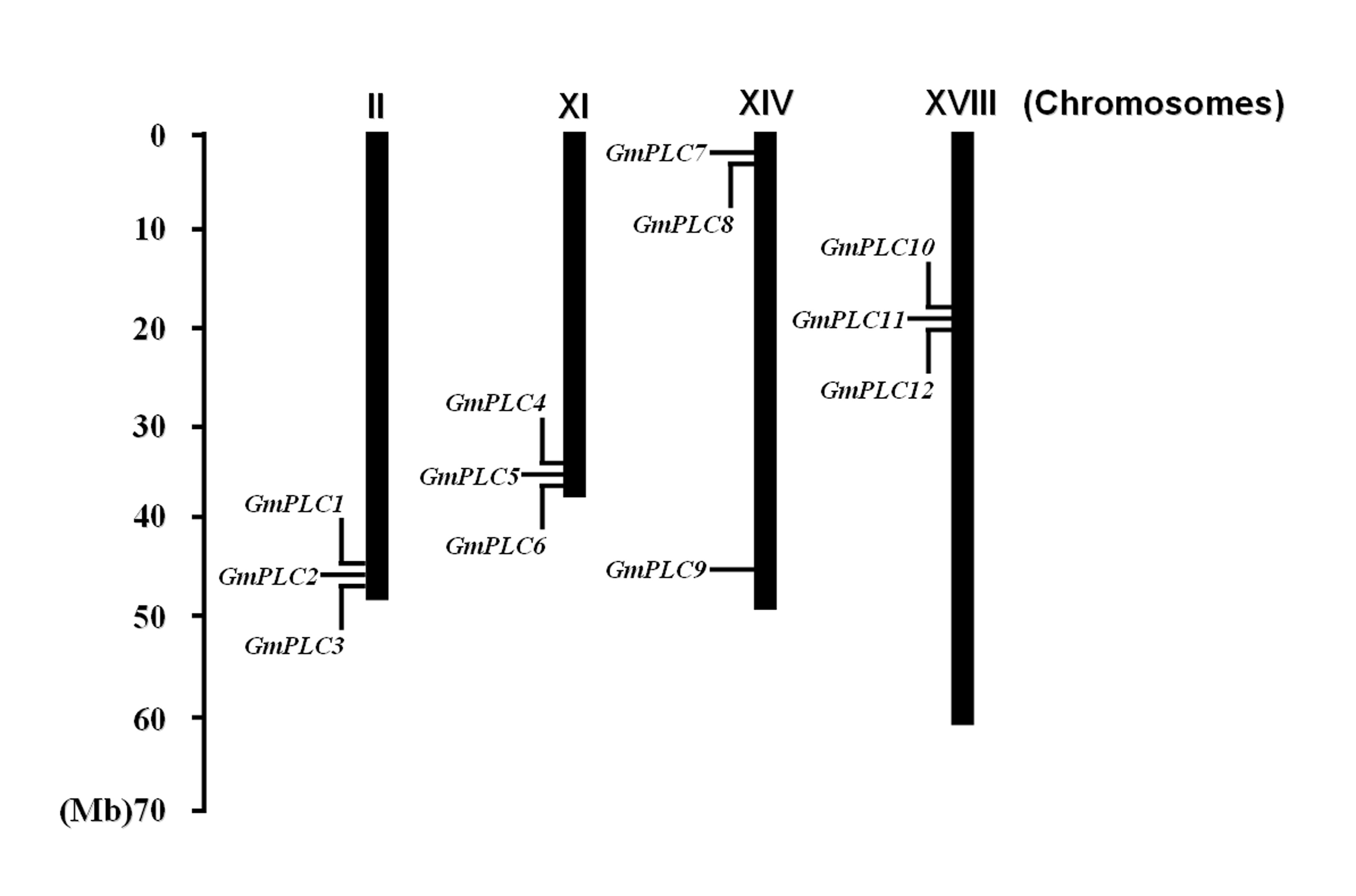

Supplement: S1 Fig — (TIF) [file pone.0138467.s004.tif]

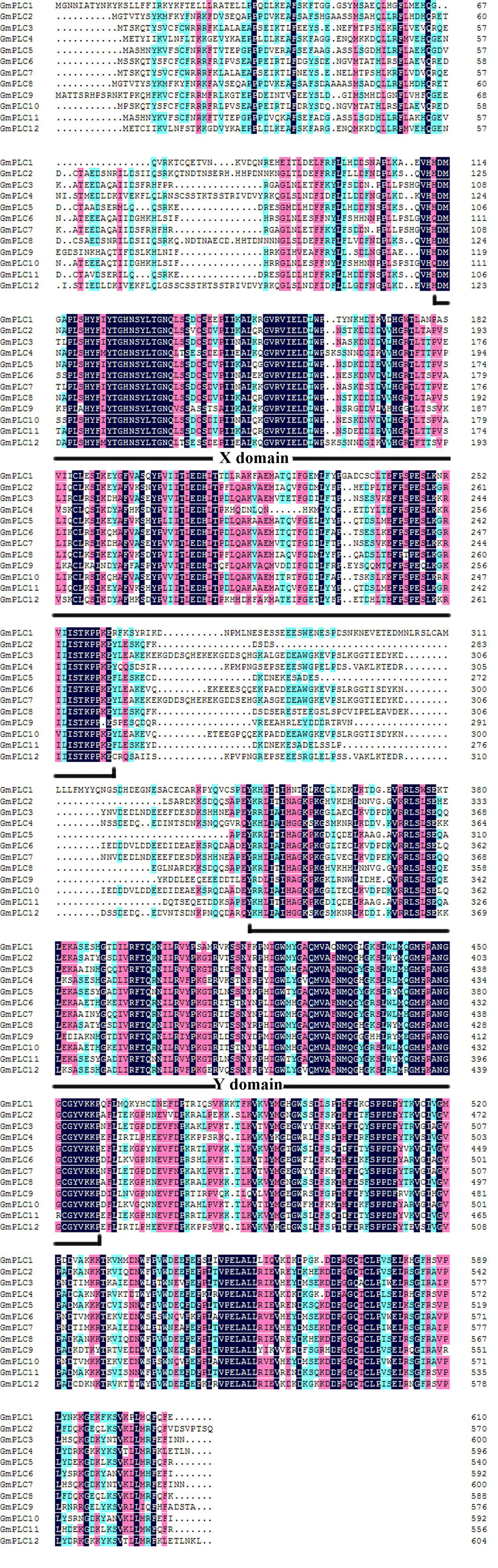

Supplement: S2 Fig — (TIF) [file pone.0138467.s005.tif]
